# Supplementary material for: Systematic Identification of Long Noncoding RNAs during Three Key Organogenesis Stages in Zebrafish
Source: Int J Mol Sci. 2024 Mar 19;25(6):3440. doi: 10.3390/ijms25063440 (PMC10970532; doi:10.3390/ijms25063440)
Supplement: Supplementary file 1 [file ijms-25-03440-s001.zip › Supplementary figure.pdf]

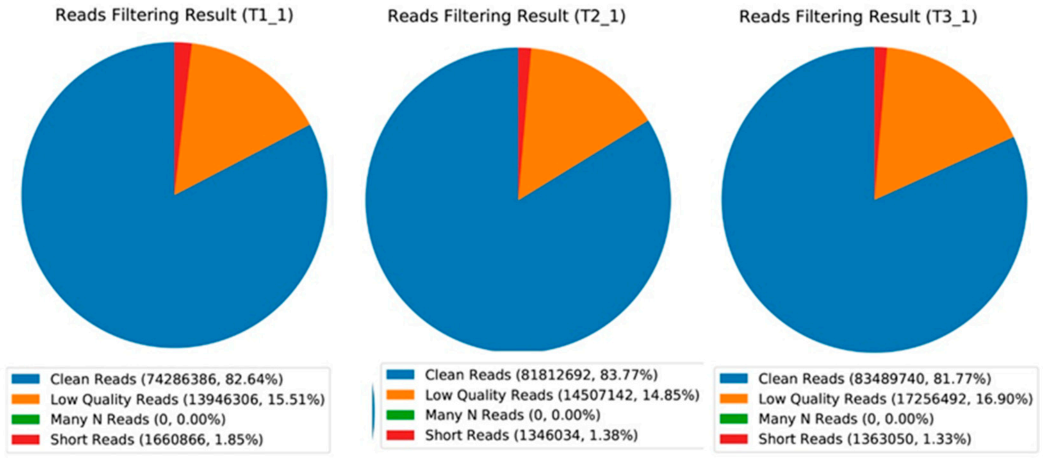

Figure S1 Distribution diagram of reads in data quality control.

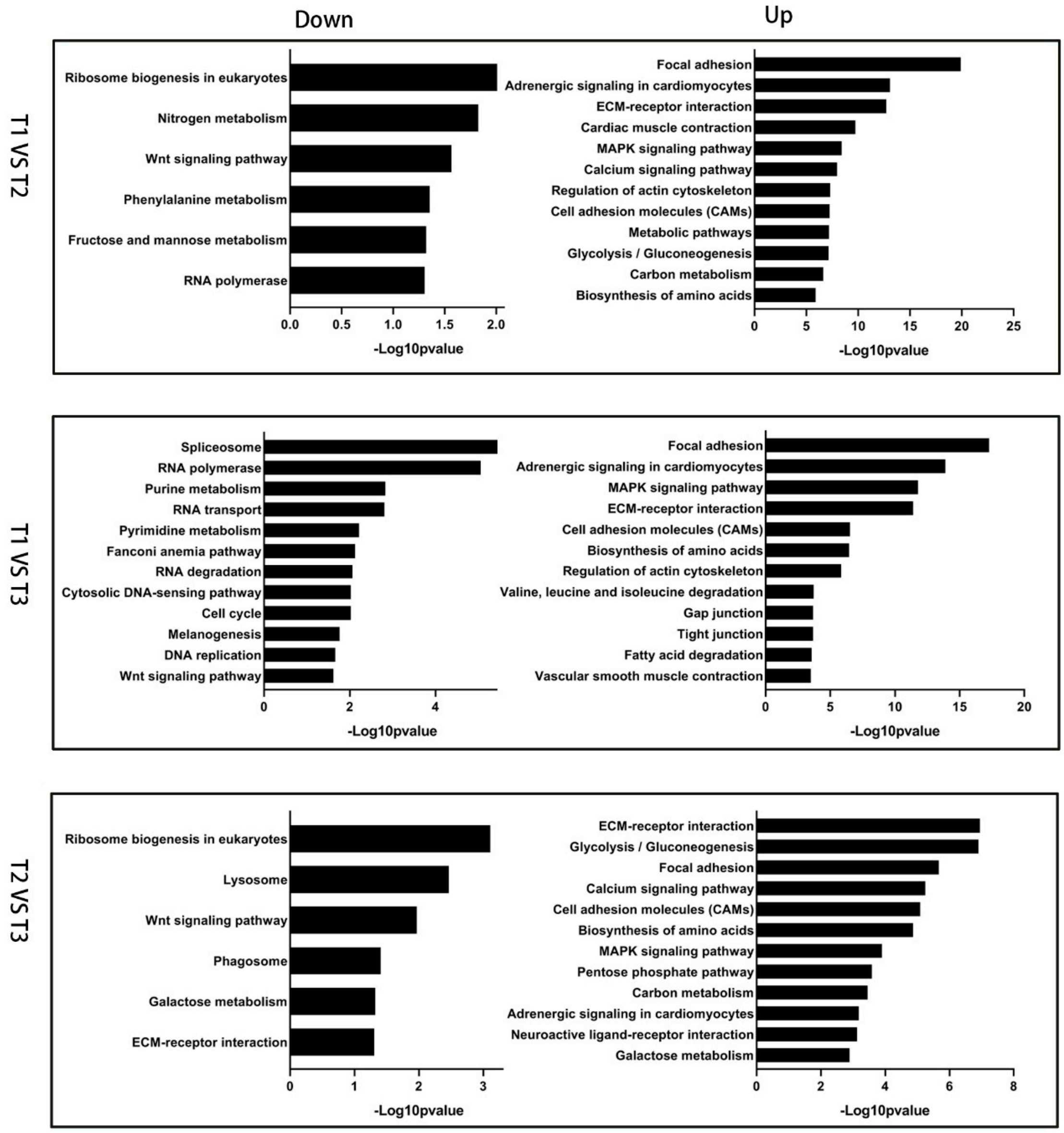

Figure S2 KEGG enrichment analysis of DEGs at different stages of zebrafish embryonic development.

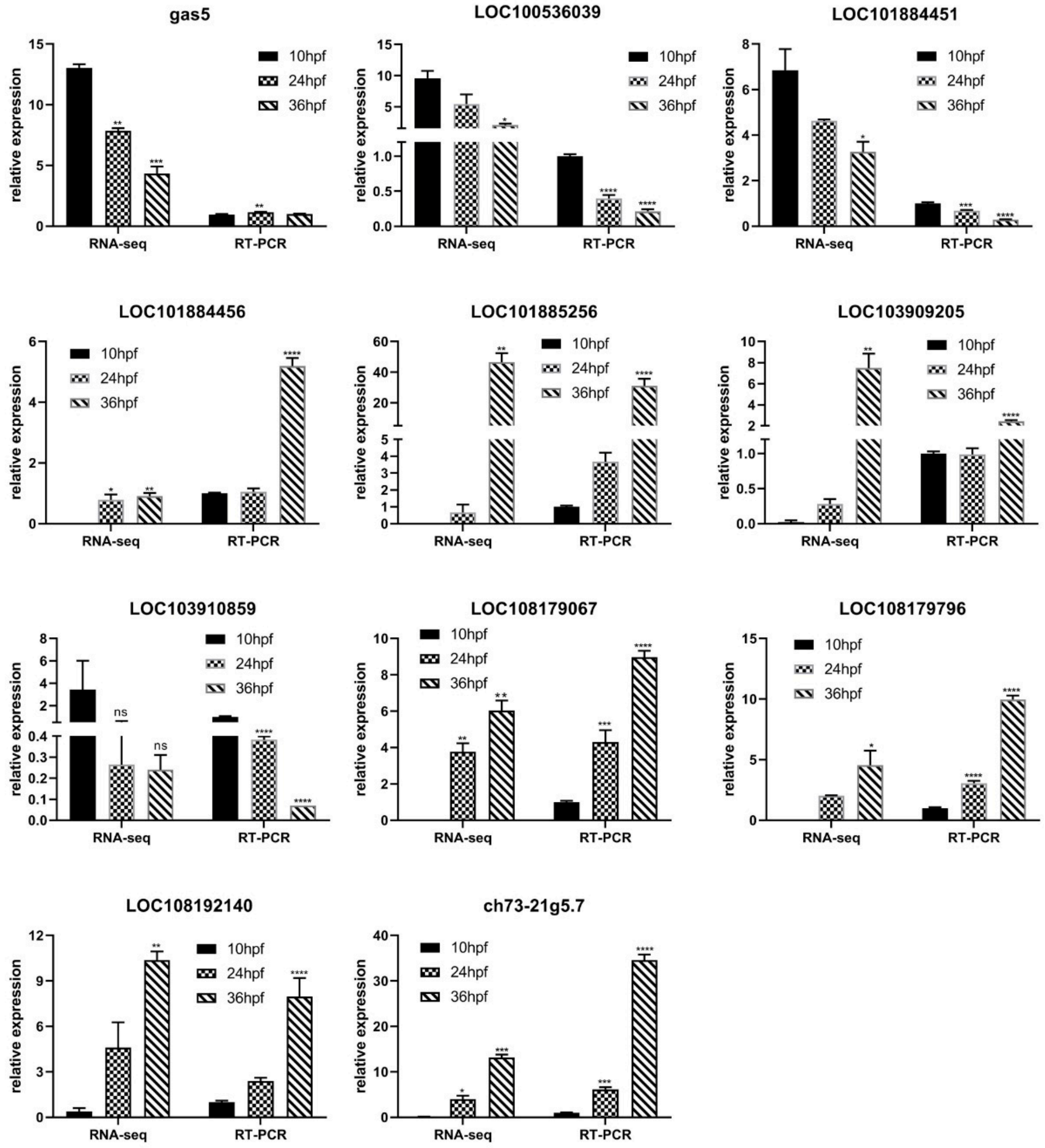

Figure S3 Verification of differentially expressed lncRNAs by qPCR at different stages of zebrafish embryonic development. The missing columns represent a relative expression of 0. Data derived from three independent experiments are shown as mean ±SEM. (*p<0.05, **p<0.01, *** p<0.001, **** p<0.0001)

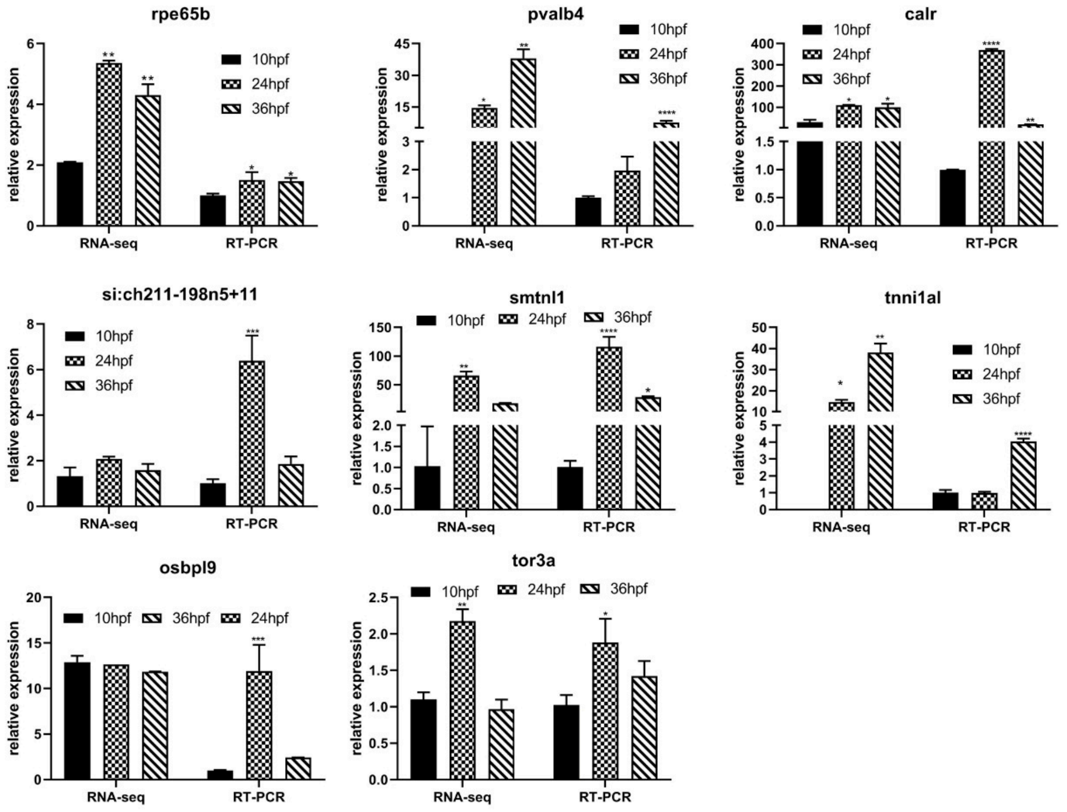

Figure S4 Verification of DEGs by qPCR at different stages of zebrafish embryonic development. Data derived from three independent experiments are shown as mean ±SEM. (**p*<0.05, ***p*<0.01, *** *p*<0.001, **** *p*<0.0001)

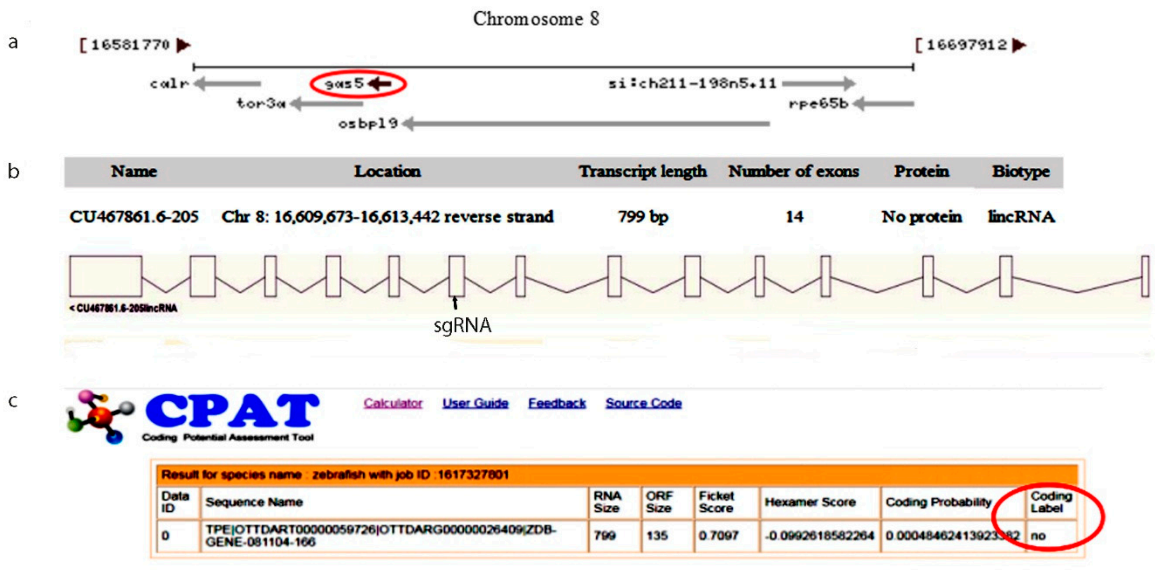

Figure S5 Bioinformatics of lncRNA *gas5*. a: The position of lncRNA *gas5* on chromosome 8 of zebrafish is marked by red circle. Red circle indicates the site of *gas5*. b: The gene architecture of lncRNA *gas5* and the site of the target site of the SgRNA. c: Coding ability prediction results are circled in red.

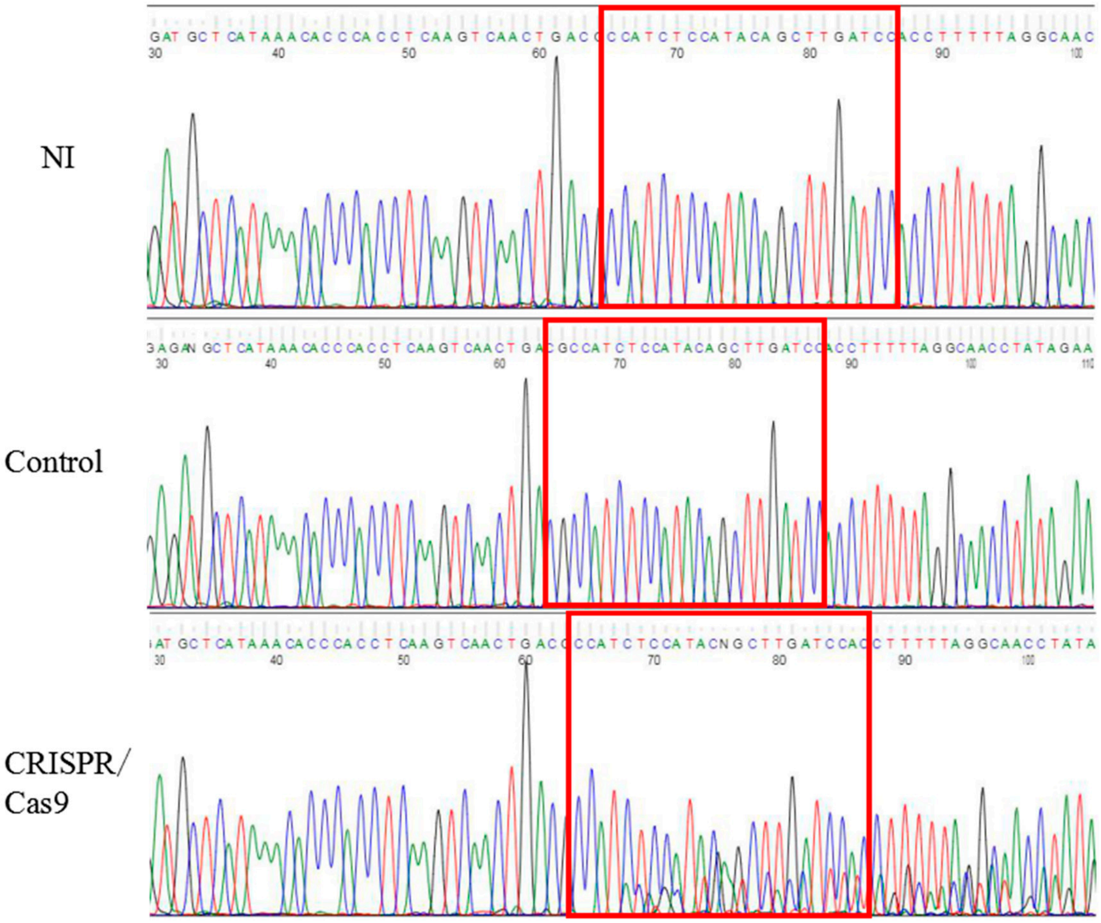

Figure S6 The validation of lncRNA gas5 knockout. ‘NI’ represents the sequencing map of 24hpf embryos with nothing injection, ‘control’ represents that of 24hpf embryos with only injection of Cas9 protein, ‘CRISPR/Cas9’ represents that of 24hpf embryos with coinjection of sgRNA and Cas9 protein. Red box is the target target site of the SgRNA.
